# TDO2-Associated Tryptophan Metabolism Correlates with Impaired Tertiary Lymphoid Structure Maturation and Reduced B Cell Class Switching in Breast Cancer

**DOI:** 10.32604/or.2026.071122

**Published:** 2026-02-24

**Authors:** Weiping Yang, Wei Xiao, Wenhao Xu, Lijun Ren, Xian Li, Junhua Yu, Ronghua Wang

**Affiliations:** 1 Department of Breast and Thyroid Surgery, Qingdao Chengyang People’s Hospital, Qingdao, 266109, China; 2 Department of Hematology, The Seventh Affiliated Hospital, Sun Yat-sen University, Guangdong, 518107, China; 3 Department of Oncology, Shanghai Medical College, Fudan University, Shanghai, 200032, China; 4 Rudong People’s Hospital/Affiliated Rudong Hospital of Xinglin College, Nantong University, Nantong, 226007, China

**Keywords:** Spatial transcriptomics, kynurenine, aryl hydrocarbon receptor (AhR), tryptophan 2,3-dioxygenase (TDO2), tertiary lymphoid structures (TLS), breast cancer, tumor immune microenvironment

## Abstract

**Background:**

Tertiary lymphoid structures (TLSs) promote antitumor immunity and predict favorable immunotherapy outcomes in breast cancer. The study aimed to investigate how Tryptophan 2,3-dioxygenase (TDO2)-associated tryptophan metabolism influences TLS maturation and B cell class switching in breast cancer.

**Methods:**

Bulk transcriptomic data from The Cancer Genome Atlas-Breast Invasive Carcinoma (TCGA-BRCA, *n* = 1055) were analyzed using Gene Set Variation Analysis (GSVA)–based metabolic scoring, immune deconvolution, and TLS quantification. Single-cell RNA sequencing (scRNA-seq, *n* = 26) and spatial transcriptomics (*n* = 1) were applied to map TDO2 expression and TLS spatial organization. Validation was performed by immunohistochemistry (*n* = 38) and multiplex immunofluorescence (*n* = 12).

**Results:**

We identified that elevated tryptophan metabolism was predominantly enriched in the Luminal A subtype and delineates an immune-cold phenotype with less immunogenicity, associated with a distinct immune-dominant cellular microenvironment, particularly enriched in T and plasma cells. High expression of the tryptophan-metabolizing enzyme TDO2 was significantly enriched in TLS-low tumors and negatively correlated with TLS maturation signatures. Functional enrichment revealed suppressed B cell class switching and attenuated C-X-C motif chemokine ligand 9 (CXCL9) expression in TLS-deficient tumors. Spatial transcriptomics and hotspot analysis demonstrated an inverse spatial correlation between TDO2 expression and TLS core components. Tumors with high tryptophan metabolism showed decreased cluster of differentiation 20 (CD20)^+^ and CXCL9^+^ cell infiltration within TLS zones. Tumors with strong TDO2–kynurenine activity displayed impaired TLS organization and attenuated humoral immunity. Conditional spatial co-occurrence modeling confirmed reduced proximity between tryptophan metabolism hotspots and TLS-related immune features.

**Conclusion:**

In conclusion, our findings suggest that TDO2-associated tryptophan metabolism is linked to impaired TLS maturation and suppressed B cell class switching in breast cancer. Targeting the TDO2-kynurenine axis may represent a promising strategy to restore TLS formation and enhance immunotherapy responsiveness in breast cancer.

## 1 Introduction

Breast cancer (BRCA) is the most commonly diagnosed malignancy and remains a leading cause of cancer-related mortality among women worldwide [1,2]. Despite significant advances in molecular classification and targeted therapies, the marked heterogeneity of breast cancer continues to pose challenges for accurate prognostication and effective treatment stratification [3,4]. In recent years, growing attention has been directed toward the tumor immune microenvironment (TIME), which plays a pivotal role in tumor progression, immune escape, and response to immunotherapy. Among the key components of TIME, tertiary lymphoid structures (TLS), ectopic lymphoid aggregates resembling secondary lymphoid organs, have emerged as both prognostic and predictive biomarkers [5–7]. In particular, TLS presence and maturation have been associated with favorable outcomes and improved responsiveness to immune checkpoint inhibitors (ICIs), especially in triple-negative and human epidermal growth factor receptor 2 (HER2)-enriched breast cancer subtypes [8,9].

TLSs are composed of spatially organized immune cell compartments, including T cell zones, B cell follicles, high endothelial venules, follicular dendritic cells, and germinal centers [6,10]. Their functional maturation is essential for the initiation and amplification of local antitumor immune responses. Mature TLSs facilitate antigen presentation, T and B cell activation, and high-affinity antibody production through germinal center formation and immunoglobulin class-switch recombination (CSR) [11–13]. However, in a substantial proportion of breast cancer cases, TLSs remain in an immature state, lacking well-defined B cell zones and class-switched immunoglobulins. This suggests the presence of tumor-intrinsic or microenvironmental factors that impair TLS development, particularly by disrupting B cell function.

Among the cellular components within TLSs, B cells play a central role in coordinating local antigen presentation, antibody production, and germinal center formation [14]. A hallmark of B cell activation and functional maturation is class-switch recombination (CSR), which facilitates the production of high-affinity IgG isotypes. Impaired CSR not only limits humoral immune responses but also reflects TLS immaturity. While prior research has focused largely on T cell and dendritic cell contributions to TLS induction, the regulation of B cell functionality, especially CSR, by tumor-derived metabolic signals remains poorly understood [15,16].

One key metabolic pathway implicated in tumor immune evasion is tryptophan catabolism [17]. Tryptophan is degraded via the kynurenine pathway, primarily through the enzymatic activity of indoleamine 2,3-dioxygenase 1 (IDO1) and tryptophan 2,3-dioxygenase (TDO2) [18,19]. Both enzymes are upregulated in various cancers and contribute to immunosuppression by generating the metabolite kynurenine, which activates the aryl hydrocarbon receptor (AhR) [20,21]. This signaling axis promotes regulatory T cell expansion, suppresses effector T cell activity, and fosters an immunosuppressive microenvironment [22,23]. While TDO2 has been implicated in immunosuppression through kynurenine-AhR signaling in other cancers, its potential role in shaping TLS maturation and B cell functional programming within the tumor immune microenvironment remains undefined [24]. We hypothesized that tumor-intrinsic TDO2 activity is associated with suppression of TLS maturation by impairing B cell class switch recombination and reducing CXCL9 expression.

In this study, we hypothesize that the TDO2, kynurenine activity biomarker, AhR axis suppresses TLS maturation in breast cancer by impairing B cell class switching. To investigate this, we performed an integrative multi-omics analysis leveraging bulk transcriptomic data from over 1000 BRCA samples in The Cancer Genome Atlas (TCGA), coupled with immune deconvolution algorithms to characterize immune infiltration patterns. We further employed single-cell RNA sequencing (scRNA-seq), pseudotime trajectory analysis, and spatial transcriptomics to dissect B cell dynamics and TLS architecture *in situ*. Spatial co-localization analyses revealed the proximity of TDO2 expression and TLS-related markers, suggesting direct spatial interference. Finally, these findings were validated through immunohistochemistry and multiplex immunofluorescence, confirming the inverse relationship between tryptophan metabolic activity and TLS maturity. Together, this study aimed to investigate the role of tumor-intrinsic TDO2 and kynurenine metabolism in shaping TLS maturation in breast cancer. Based on multi-omics and spatial analyses, we tested three specific hypotheses: (1) TDO2 expression spatially anti-correlates with TLS markers and mature germinal center–like structures; (2) enhanced tryptophan metabolism is associated with reduced B cell class switch recombination; and (3) TDO2 activity correlates with decreased CXCL9 expression within TLS-enriched zones.

## 2 Methods and Materials

### 2.1 Samples Collection and Processing

Gene expression data (bulk RNA-seq) and corresponding clinical information for BRCA cases were retrieved from the TCGA portal (https://portal.gdc.cancer.gov/). After filtering, 1055 patient samples were retained for downstream analysis. Expression levels across the three datasets were measured in transcripts per million (TPM) and log2(TPM + 1) normalization was applied to maintain consistency across data. Low-abundance transcripts, defined as those with TPM values below 1 in more than 90% of samples, were excluded to minimize background noise. Additionally, cases lacking matched gene expression and clinical data, or missing follow-up information, were excluded to reduce analytical bias. Overall survival (OS) was used as the primary clinical endpoint.

A total of 38 human breast cancer specimens were included for immunohistochemistry (IHC) and multiplex immunofluorescence (mIF) validation. Samples were collected from Qingdao Chengyang People’s Hospital. All procedures involving human participants were conducted in accordance with the Declaration of Helsinki and were approved by the Institutional Review Board of Qingdao Chengyang People’s Hospital (Approval No. 2024LCSY086). Written informed consent was obtained from all patients prior to sample collection.

### 2.2 Identification of Different Tryptophan Patterns across BRCA

To investigate the metabolic reprogramming of amino acid pathways in BRCA, Gene Set Variation Analysis (GSVA) was performed based on the R package ‘GSVA’ (v1.44.5) [25]. Tryptophan metabolism-related gene sets were curated from the Kyoto Encyclopedia of Genes and Genomes (KEGG) database (https://www.kegg.jp/). Samples were stratified into high and low tryptophan metabolism groups based on median value. To further characterize the biological functions associated with each metabolic pattern, Gene Ontology (GO) enrichment analysis was performed using the clusterProfiler R package (version 4.6.2). Enriched GO terms for biological process, molecular function, and cellular component categories were identified based on a Benjamini-Hochberg-adjusted *p* < 0.05.

### 2.3 Comparison of Diverse Immune Infiltration Estimation

To assess the immune landscape of breast cancer samples, we utilized the ‘IOBR’ (v2.0.0) R package [26], an integrative platform for immuno-oncology biomarker discovery. Gene expression matrices were first normalized and input into IOBR to calculate immune cell infiltration scores across multiple algorithms, including Estimate, CIBERSORT, EPIC, quanTIseq, MCP-counter, TIMER, and xCell. Heatmaps were generated to visualize the immune infiltration profiles across high and low tryptophan metabolism activities.

### 2.4 Single-Cell RNA Sequencing Analysis

Single-cell transcriptomic data were obtained from the Gene Expression Omnibus (GEO) database (https://www.ncbi.nlm.nih.gov/geo/ (accessed on 30 July 2025)) (GSE176078, *n* = 26) [27]. Data preprocessing was performed using the ‘Seurat’ (v4.4.0) package [28]. Cells with fewer than 200 or more than 2500 detected genes, or those with over 10% mitochondrial gene content, were excluded to ensure data quality (Table S1). To mitigate the influence of cell cycle heterogeneity, the CellCycleScoring function was employed to assign each cell to a cell cycle phase. The gene expression matrix was processed through log-transformation and normalization utilizing the LogNormalize approach implemented by the NormalizeData function. To identify genes with high variability, the FindVariableFeatures function was applied with the “vst” method, and the 2000 most variable genes were selected for further analysis.

Dimensionality reduction and visualization of single-cell RNA sequencing (scRNA-seq) data were performed using Uniform Manifold Approximation and Projection (UMAP) and t-distributed Stochastic Neighbor Embedding (t-SNE) algorithms, implemented in the Seurat R package (version 4.3.0, https://satijalab.org/seurat/ (accessed on 30 July 2025)). Both algorithms were applied to log-normalized gene expression matrices after principal component analysis (PCA) using the top 30 principal components. UMAP was used for global visualization of cellular heterogeneity, while t-SNE was employed for fine-grained clustering of cell subpopulations. Cell identities were annotated based on canonical marker genes and verified by cluster-specific expression profiles. PCA was then performed using the RunPCA function, with the top 50 principal components retained for dimensionality reduction. Clustering was performed using a shared nearest neighbor (SNN) modularity optimization algorithm via FindNeighbors and FindClusters, respectively. Cell populations were manually annotated based on canonical marker gene expression, as reported in prior literature [27,29,30].

### 2.5 Pseudotime Trajectory Analysis

All B cells were extracted for trajectory analysis using the ‘Monocle2’ R package [31]. Highly variable genes were identified with dispersionTable to define the trajectory feature space. Dimensionality reduction was performed via the DDRTree algorithm, and cell state transitions were inferred using reduceDimension. The pseudotime trajectory and gene dynamics were visualized with plot_cell_trajectory highlighting functional changes in B cells over developmental progression.

### 2.6 Spatial Transcriptome Data Collection and Preprocessing

Spatial transcriptome (ST) data (V1_Breast_Cancer_Block_A_Section_2_spatial) of breast cancer were retrieved from 10x Genomics official website (https://www.10xgenomics.com/). All preprocessing and analytical workflows were conducted in R using the ‘Seurat’ package. For normalization and dataset harmonization, the SCTransform (SCT) pipeline was implemented, which utilized key functions including SelectIntegrationFeatures, PrepSCTIntegration, FindIntegrationAnchors, and IntegrateData. Unsupervised clustering was performed to delineate spatially distinct tissue domains. To determine cell type identities, hematoxylin and eosin (HE) staining outcomes were combined with the assessment of the most variable genes within individual clusters. SpatialDimPlot and SpatialFeaturePlot functions were employed in combination to depict gene expression distributions across spatial coordinates.

### 2.7 Spatial Deconvolution Using Robust Cell Type Decomposition (RCTD)

Spatial deconvolution was performed using Robust Cell Type Decomposition (RCTD) to infer cell-type composition within each spatial transcriptomics spot based on a reference scRNA-seq dataset. After normalization and preprocessing, a SpatialRNA object was constructed and input into the create. RCTD (V1.3.0) function [32]. Deconvolution was performed in multiple modes, and cell type proportions were normalized. Results were visualized to show spatial distribution of inferred cell types.

### 2.8 Spatial Co-Occurrence Analysis of Tryptophan Metabolism and TLS-Associated Markers

To explore spatial relationships between tryptophan metabolism and TLS-related features, we performed co-occurrence and proximity analysis using ‘Squidpy’ (version 1.2.3). Based on smoothed spatial transcriptomics data, we first defined regions of high expression for TLS markers (e.g., CXCL9), high endothelial venules (HEVs), and key enzymes of tryptophan metabolism (e.g., TDO2, IDO1). Co-occurrence scores were computed using a conditional probability model across increasing distances (0–6000 μm), classifying spatial spots into combinations such as CXCL9^+^/TDO2^+^, CXCL9^−^/TDO2^+^, etc. These curves reflect the likelihood of proximity or avoidance between metabolic activity and immune structures. This approach highlights the spatial dependency between immunoregulatory tryptophan metabolism and TLS components, suggesting potential metabolic constraints on TLS maturation. To statistically assess whether observed co-occurrence exceeded random expectations, we performed permutation testing (*n* = 1000) by shuffling spatial labels and recomputing co-occurrence scores under the null. We derived empirical *p*-values and 95% confidence intervals (CIs) for each cell pair type (e.g., CXCL9^+^ vs. TDO2^+^), and evaluated whether observed values fell outside the null distribution.

### 2.9 Hotspot Analysis

We used the Hotspot module to detect spatially variable genes based on smoothed expression data. Tryptophan metabolism genes and TLS-related markers (e.g., CXCL9, HEV markers) were analyzed to identify local expression hotspots. This revealed spatial clusters where metabolic and immune signals co-localized, highlighting regions potentially involved in TLS maturation [32].

### 2.10 Leave-One-Out (LOO) Analysis

To evaluate the robustness and sample-specific influence on GC-like B cell frequency estimation, a leave-one-out (LOO) analysis was performed. For each iteration, one patient sample was systematically removed from the cohort (*n* = 26), and the mean GC-like B cell frequency was recalculated across the remaining samples. The absolute frequency difference between the full dataset and each leave-one-out subset (High-Low) was computed to quantify the influence of individual samples on group-level enrichment. Samples yielding the largest deviations were identified as having the strongest impact on GC-like B cell frequency patterns. All computations were performed using custom R scripts (version 4.3.1).

### 2.11 Differential Expression and Functional Enrichment Analysis

Differentially expressed genes (DEGs) between TLS-high and TLS-low regions were identified using a negative binomial framework with Benjamini–Hochberg correction. Unless otherwise specified, gene-level significance was defined as FDR-adjusted *p* < 0.05 with an effect-size filter of |log_2_FC| > 0.5. Results were visualized in volcano plots using these thresholds. For pathway analysis, we performed (i) over-representation tests (Benjamini–Hochberg [BH]-adjusted *p* < 0.05) and (ii) Gene Set Enrichment Analysis (GSEA), for which gene-set significance was defined as FDR q < 0.25, consistent with established GSEA practice [33]. Where noted, we conducted sensitivity analyses applying a more stringent effect-size threshold (|log_2_FC| > 0.75) to confirm robustness of pathway-level findings. Single-sample Gene Set Enrichment Analysis (ssGSEA) was performed using the GSVA R package (version 1.44.5, Bioconductor, https://bioconductor.org/packages/GSVA/ (accessed on 30 July 2025)). This algorithm calculates an enrichment score for each gene set in individual samples by ranking genes based on their expression and integrating their empirical cumulative distribution functions. Gene signatures representing metabolic pathways, immune-related processes, and TLS maturation were derived from the Kyoto Encyclopedia of Genes and Genomes (KEGG) and prior literature.

### 2.12 Hematoxylin and Eosin (HE) Staining

Formalin-fixed, paraffin-embedded (FFPE) breast tumor sections (4 µm) were deparaffinized in xylene, rehydrated through graded ethanol, and stained with Mayer’s hematoxylin(1x) for 3–5 min, followed by 0.5% eosin Y counterstaining for 30–60 s. Slides were dehydrated, cleared in xylene, and mounted with resin. Each batch included quality-control slides to ensure consistent nuclear-cytoplasmic contrast. Stained sections were reviewed by two independent pathologists blinded to molecular data to confirm tumor histology, grade, and stromal features. The presence and maturation of tertiary lymphoid structures (TLSs) were evaluated based on organized lymphoid aggregates with defined B/T-cell zones and germinal center-like morphology. Discrepancies were resolved by consensus.

### 2.13 Immunohistochemistry and Scoring Assays

Formalin-fixed paraffin-embedded (FFPE) breast cancer sections (*n* = 38) were used for IHC staining. Tumor specimens were randomly selected from the study cohort, stratified by TLS status. Immunohistochemical (IHC) staining was performed on breast cancer tissue sections to evaluate CXCL9 expression. Kynurenine pathway activity was assessed using Indoleamine 2,3-dioxygenase (IDO) staining with the IDO (D5J4E™) Rabbit mAb (1:200 dilution, Cat. no. 86630, *Cell Signaling Technology*, Inc., 3 Trask Lane, Danvers, MA, USA). The antibody recognizes endogenous levels of total IDO (IDO-1, INDO) protein. The intensity of IHC staining was quantified using a semi-quantitative scoring system (IHCscore) with image acquisition and analysis performed using the Aperio ImageScope digital pathology system (version 12.4.3, Leica Biosystems, 1700 Leider Lane, Buffalo Grove, IL, USA). The scoring included two components: (1) Proportion score, based on the percentage of positively stained cells (0 = none; 1 = 1%–25%; 2 = 26%–50%; 3 = 51%–75%; 4 = 76%–100%); and (2) Intensity score, based on staining strength (0 = negative; 1 = weak; 2 = moderate; 3 = strong). The final IHC score was calculated by multiplying the proportion score by the intensity score.

### 2.14 Multiplex Immunofluorescence (mIF) Staining Analysis

Multiplex immunofluorescence (mIF) was performed on formalin-fixed, paraffin-embedded (FFPE) breast cancer sections using the Opal™ Polaris 7-Color IHC Kit (Akoya Biosciences, Inc., 100 Campus Drive, Marlborough, MA, USA) to evaluate the spatial co-localization of immune and metabolic markers. Primary antibodies included CD3 (1:200, Cat. no. ab16669, Abcam, Cambridge, UK), CD20 (1:150, Cat. no. ab9475, Abcam), CXCL9 (1:100, Cat. no. DF6015, Affinity Biosciences, Cincinnati, OH, USA), and TDO2 (1:200, Cat. no. 12216-1-AP, Proteintech Group, Rosemont, IL, USA). Antigen retrieval was re-performed after each staining cycle to eliminate prior antibody complexes. The study utilized Opal dyes: 620 for CD3, 570 for CD20, 690 for CXCL9, and 520 for TDO2. Nuclear visualization was achieved with DAPI staining [34,35].

Whole-slide imaging was carried out using the Vectra Polaris Imaging System (Akoya Biosciences, Marlborough, MA, USA) under consistent exposure conditions. Quantitative image assessment was performed using the HALO platform (Indica Labs, Albuquerque, NM, USA). Nuclei segmentation and marker-based phenotyping identified CD3^+^ T cells, CD20^+^ B cells, and TDO2^+^ tumor cells. Spatial proximity analysis defined co-localization when cell centroids were within 50 μm. Image scoring was blinded to TLS status, and reproducibility was confirmed by independent reviewers.

### 2.15 Criteria for TLS Maturation

To distinguish between mature and immature TLSs, we applied an integrated set of transcriptomic, cellular, and histological criteria. TLS regions were first identified based on spatial clustering of TLS signature scores and hotspot analysis. TLSs were defined as mature if they exhibited: (1) enrichment of canonical TLS-associated cytokines and chemokines (CXCL9, CXCL13, TNFSF13, RORA) together with elevated expression of germinal center–related markers (AICDA, CD38, IgG); (2) presence of germinal center–like B cell subsets and plasmablasts as determined by scRNA-seq and pseudotime trajectory analysis; and (3) histological features consistent with organized CD3^+^ T cell zones and CD20^+^ B cell follicles, confirmed by multiplex immunofluorescence, along with higher CXCL9 expression. TLSs lacking these molecular and architectural features, particularly the absence of class-switched B cell subsets and organized germinal center–like zones, were classified as immature.

### 2.16 Statistical Analysis

All statistical analyses were performed in R 4.2.1 and Python 3.7.0. For categorical variables (e.g., TLS-high vs. TLS-low frequencies), we used Fisher’s exact test. For continuous variables, Student’s *t*-tests were applied when assumptions of normality (tested by the Shapiro–Wilk test) and equal variance (tested by the Levene’s test) were met; otherwise, the Mann–Whitney U test was used. For comparisons involving more than two groups, the Kruskal–Wallis test was employed with appropriate post hoc adjustments. The software and version used in this study are shown in Table S2.

## 3 Results

### 3.1 Tryptophan Metabolism Activity Associated with Distinct Immune Landscapes and Clinical Features in Breast Cancer

**Figure 1:**
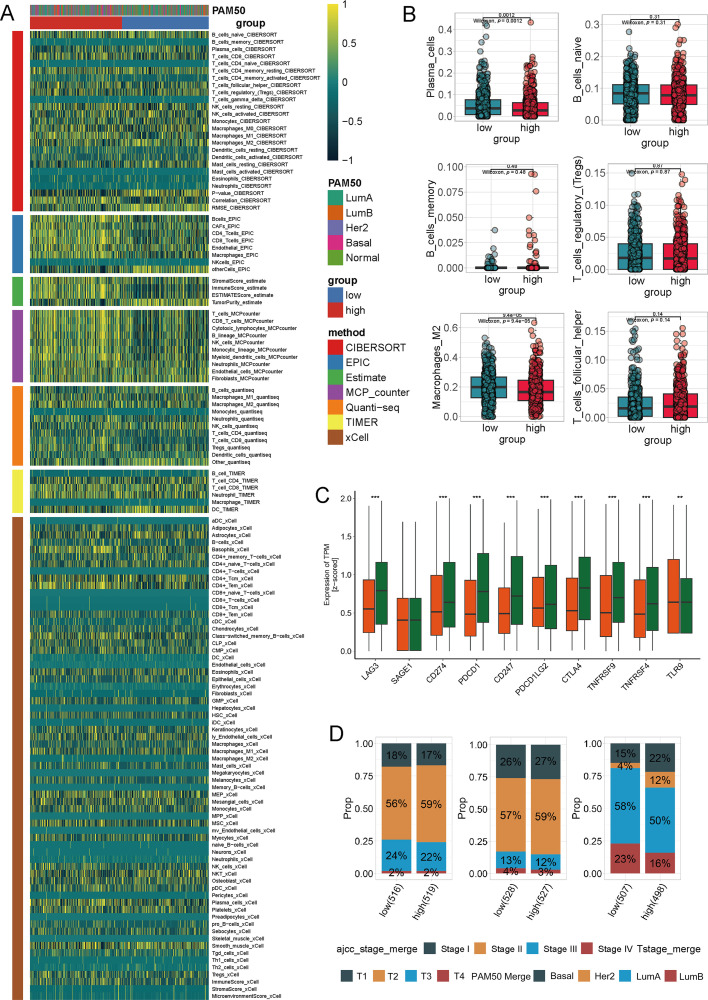
Immune profile and clinical characteristics associated with tryptophan metabolism in breast cancer. (**A**) The heatmap displays the immune cell infiltration in breast cancer samples with high and low tryptophan metabolism scores, estimated using seven computational methods (CIBERSORT, EPIC, Estimate, MCP-counter, quanTIseq, TIMER,xCell). Samples are labeled with PAM50 molecular subtypes and tryptophan metabolism groups. (**B**) Difference of specific immune cell subpopulations among the high and low tryptophan metabolism groups, including plasma cells, naive B cells, memory B cells, regulatory T cells (Treg), follicular helper T cells (Tfh), and M2-like macrophages. (**C**) The expression of key immune checkpoint-related genes stratified by tryptophan metabolism activity (e.g., LAG3, SAGE1, CD274/PD-L1, PDCD1/PD-1, CTLA4, TNFRSF4, TLR8). (**D**) The bar chart shows the proportional distribution of clinical and molecular subgroups (American Joint Committee on Cancer [AJCC] staging, T staging, and PAM50 subtypes) within the high and low tryptophan metabolism groups (^**^p < 0.01, ^***^p < 0.001)

To comprehensively investigate the link between tryptophan metabolism and TLS maturation in breast cancer, we integrating bulk RNA-seq, single-cell RNA-seq, spatial transcriptomics, and multiplex immunofluorescence (mIF) validation (Fig. S1). To investigate the immunological implications of tryptophan metabolism in breast cancer, we first stratified TCGA breast cancer samples into high and low tryptophan metabolism groups based on ssGSEA scores. A comprehensive immune deconvolution analysis using seven established algorithms (CIBERSORT, EPIC, Estimate, MCP-counter, quanTIseq, TIMER, and xCell) revealed marked differences in immune cell infiltration patterns between the two groups (Fig. 1A, Table S3).

The high-tryptophan group showed overall reduced immune diversity and lower abundance of multiple effector lineages together with lower expression of immune-activation/checkpoint markers (e.g., LAG3, CD274/PD-L1, PDCD1/PD-1, CTLA4, TNFRSF4), consistent with an immune-cold/low-immunogenic milieu (Fig. 1A–C). Within this globally suppressed context, we observed a relative enrichment of T cells and plasma cells compared with other immune compartments; however, this did not translate into higher immune activation, as reflected by the reduced checkpoint/activation gene expression. Clinically, the high-tryptophan group was enriched for Luminal A tumors and earlier stage categories (Fig. 1D), while no significance was found. Collectively, these data indicate that elevated tryptophan metabolism marks an immune-cold state with attenuated immune activation and reduced lineage breadth, despite a relative over-representation of specific subsets (T cells and plasma cells).

### 3.2 Single-Cell Profiling Reveals Cellular Landscape and Compositional Shifts Associated with Tryptophan Metabolism

To further delineate the cellular basis underlying this metabolic-immune association, we performed single-cell transcriptomic analysis to construct a high-resolution cellular atlas and evaluate the composition of the breast cancer microenvironment under different tryptophan metabolic states.

**Figure 2:**
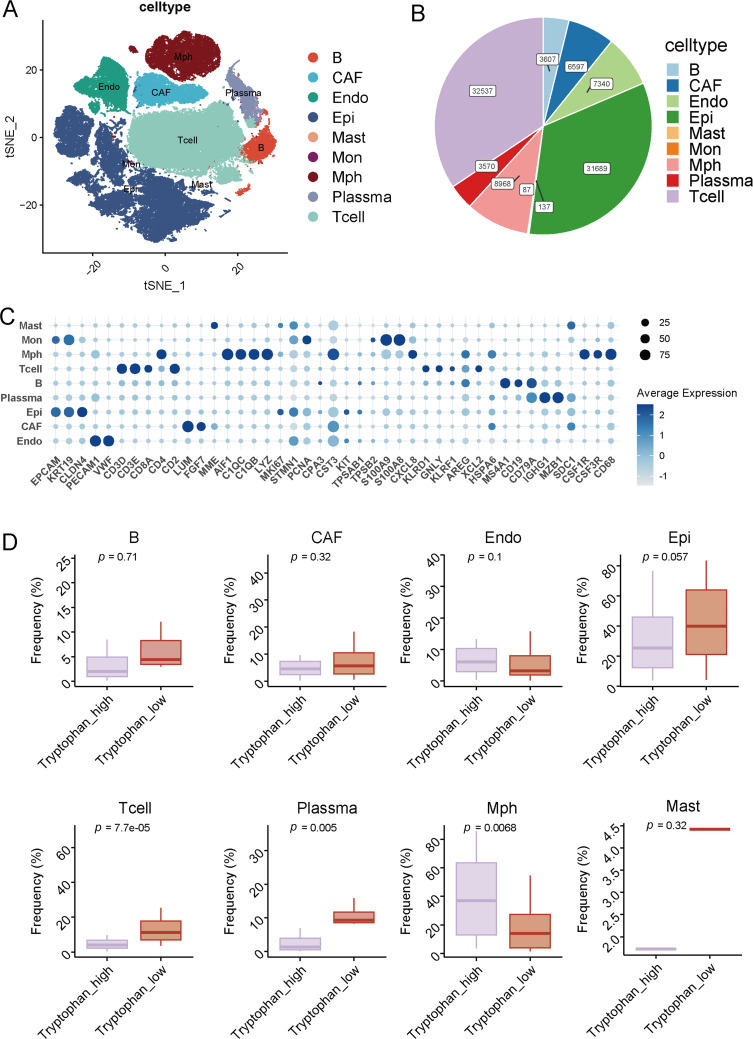
Single-cell atlas and cellular composition related to tryptophan metabolic status. (**A**) The t-SNE plot illustrates the clustering of major cell types identified in the single-cell transcriptomic dataset of breast cancer. Cells are annotated into nine distinct cell groups, including B cells, T cells, epithelial cells (Epi), endothelial cells (Endo), cancer-associated fibroblasts (CAF), mast cells, monocytes (Mon), macrophages (Mph), and plasma cells. (**B**) The pie chart displays the proportion of each annotated cell type across the entire dataset. (**C**) The dot plot represents the average expression and expression rates of typical marker genes in each cell type. (**D**) The box plot shows the relative abundance of each cell type between the high-tryptophan and low-tryptophan groups

After quality control (Fig. S2), we analyzed a total of 107,149 single cells from breast cancer samples and identified nine major cell types using canonical marker gene expression and unsupervised clustering. These populations included B cells, T cells, epithelial cells (Epi), endothelial cells (Endo), cancer-associated fibroblasts (CAF), mast cells, monocytes (Mon), macrophages (Mph), and plasma cells (Fig. 2A). The overall proportion of each cell population is depicted in the pie chart, with T cells (23.6%), epithelial cells (21.7%), and CAFs (18.3%) constituting the predominant subsets (Fig. 2B). Marker-based validation was conducted using genes such as CD79A (B cells), CD3D (T cells), EPCAM (epithelial cells), PECAM1 (endothelial cells), ACTA2 (CAFs), TPSAB1 (mast cells), CD14 (monocytes), CD68 (macrophages), and IGHG1 (plasma cells) (Fig. 2C).

We then evaluated the relative abundance of these populations between metabolic groups characterized by high and low tryptophan activity. Notably, T cells were more prevalent in the high-tryptophan group, implying enhanced recruitment or retention of lymphocytes in metabolically active tumors (Fig. 2D). Plasma cells and macrophages also showed higher frequencies in the tryptophan-high group (*p* = 0.005 and *p* = 0.0068, respectively), potentially reflecting ongoing immune activation and humoral responses. In contrast, epithelial cell proportion tended to be higher in the tryptophan-low group (*p* = 0.057), indicative of reduced immune infiltration. Other populations, including B cells, CAFs, endothelial cells, monocytes, and mast cells, did not show significant changes between the two groups. These findings indicate that tumors with elevated tryptophan metabolism are associated with a distinct immune-dominant cellular microenvironment, particularly enriched in T and plasma cells, which may contribute to enhanced TLS formation and maturation.

### 3.3 Single-Cell Transcriptomics Reveals the Heterogeneity and Differentiation Trajectory of B Cells under Distinct Tryptophan Metabolic States in Breast Cancer

**Figure 3:**
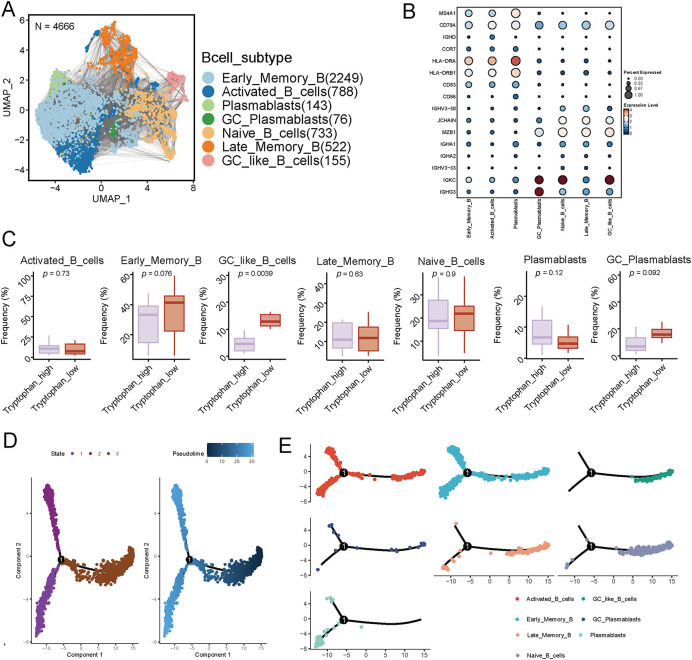

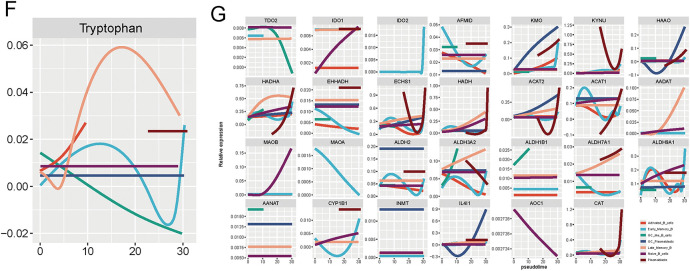
Single-cell transcriptomics reveals the heterogeneity of B cells in breast cancer and their differentiation trajectory characteristics under tryptophan metabolic states. (**A**) The UMAP plot displays 4666 B cells extracted from breast cancer samples, classified into seven subgroups based on transcriptional features: Early Memory B cells (Early_Memory_B), Activated B cells (Activated_B_cells), Naive B cells (Naive_B_cells), Late Memory B cells (Late_Memory_B), Germinal Center-like B cells (GC_like_B_cells), Germinal Center Plasmablasts (GC_Plasmablasts), and Plasmablasts (Plasmablasts). (**B**) The dot plot illustrates the expression of typical marker genes in each B cell subgroup, validating subgroup identities. (**C**) The box plot compares the frequency distribution of each B cell subgroup under different tryptophan metabolic states. (**D**) Pseudotime analysis using Monocle shows the differentiation trajectory of B cells (the left image represents state classification, while the right image depicts the pseudotime progression). (**E**) The B cell clustering results are mapped onto the pseudotime trajectory, revealing the dynamic transition relationships among subgroups during the developmental process. (**F**) The overall trend of tryptophan metabolism pathway activity along the pseudo-time axis. (**G**) Expression of TDO2, IDO1, KMO, and KYNU, during the B cell differentiation process suggests that B cells undergo metabolic reprogramming as they mature

To investigate the heterogeneity and differentiation dynamics of B cells in the tumor immune microenvironment of breast cancer, we performed scRNA-seq analysis on tumor-infiltrating B cells. A total of 4666 high-quality B cells were identified and visualized using UMAP dimensionality reduction. The analysis identified seven transcriptionally distinct B cell subtypes: Naive B cells, Early Memory, Activated, and Late Memory B cells, GC-like B cells, Germinal Center-derived plasmablasts, and conventional plasmablasts (Fig. 3A). Annotation of these populations was guided by established marker gene profiles, as illustrated in the dot plot (Fig. 3B), supporting the accuracy of the clustering.

Next, we examined the distribution of these B cell subsets across tumors stratified by different tryptophan metabolic activity levels. Interestingly, tumors with high tryptophan metabolism presented significantly reduced frequency of GC-like B cells and plasmablasts, subpopulations essential for effective humoral responses and TLS maturation, while naive and early memory B cells were relatively enriched (Figs. 3C and S3). This observation suggests that enhanced tryptophan metabolism may impede the terminal differentiation of B cells in the TIME. To further characterize B cell developmental trajectories, we performed pseudotime analysis using Monocle. The trajectory revealed a continuum of B cell states, transitioning from naive and early memory B cells toward activated and GC-like phenotypes, ultimately differentiating into plasmablasts (Fig. 3D,E). This developmental path aligns with canonical B cell maturation processes observed during adaptive immune responses.

We then assessed the dynamic activity of the tryptophan metabolism pathway along the pseudotime axis. Notably, pathway activity gradually increased during early stages of B cell maturation and plateaued or declined as cells progressed toward GC and plasmablast stages (Fig. 3F). Additionally, expression profiles of key metabolic enzymes, including TDO2, IDO1, KMO, and KYNU, varied dynamically across the differentiation timeline (Fig. 3G), suggesting that B cells undergo progressive metabolic reprogramming in the context of tryptophan metabolism during their maturation. Among them, TDO2 expression prominently increased during early-to-intermediate stages, implicating it as a potential regulator of B cell fate decisions.

Collectively, these results highlight the plasticity of tumor-infiltrating B cells in breast cancer and suggest that enhanced tryptophan metabolism may skew B cell developmental programs, potentially impeding germinal center formation and antibody-producing cell differentiation.

### 3.4 Tryptophan Metabolism Reshapes Immune Cell Composition and TLS-Associated Signaling within the Tumor Spatial Landscape

**Figure 4:**
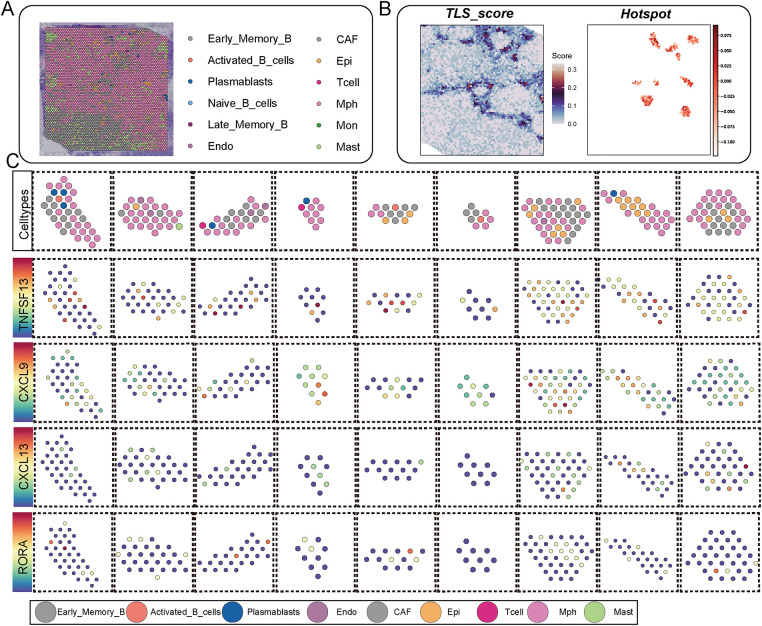

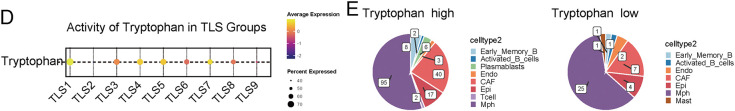
The impact of tryptophan metabolism activity on the immune cell composition and key signaling expression patterns in TLS regions. (**A**) The spatial transcriptomic slices of breast cancer were annotated for cell types using spatial deconvolution methods, illustrating the spatial distribution of different B cell subpopulations and immune cells. (**B**) The left panel shows a spatial distribution heatmap of TLS scores in spatial images, while the right panel presents hotspot areas identified by the Hotspot module with high TLS signaling. (**C**) The dot plot displays the spatial expression patterns of key signaling factors in TLS (TNFSF13, CXCL9, CXCL13, RORA) across different cell types, arranged by various subpopulations in TLS regions. In each subplot, color represents the average expression level, and dot size represents the expression proportion. (**D**) Activities of tryptophan across different TLS regions. (**E**) The change in immune cell composition ratios in TLS regions under high (Tryptophan_high) and low (Tryptophan_low) tryptophan metabolism conditions

Building on these observations, we next explored how tryptophan metabolism influences the immune cell landscape and TLS-related signaling patterns within the spatial context of breast cancer tissue. Spatial transcriptomic analysis combined with spatial deconvolution methods allowed us to annotate diverse immune cell types, including distinct B cell subpopulations, within spatial slices of breast cancer specimens (Fig. 4A, Table S4). TLS regions were identified by high TLS signature scores and further confirmed by hotspot analysis, revealing discrete immunologically active niches enriched in B cell aggregates (Fig. 4B, Table S5). We then assessed the spatial expression of canonical TLS-related cytokines and chemokines, including *TNFSF13, CXCL9, CXCL13*, and *RORA*, across various immune cell types within TLS regions. These signaling factors exhibited cell-type-specific expression patterns, underscoring the coordinated molecular interactions that sustain TLS architecture and function (Fig. 4C).

Notably, when stratifying TLS regions by tryptophan metabolism activity, we observed significantly altered signaling profiles and immune compositions. Regions with high tryptophan activity demonstrated a reduction in GC-like B cells and T follicular helper cells, alongside diminished expression of CXCL13 and RORA, both of which are critical for TLS maturation (Fig. 4D,E). The results imply that increased tryptophan metabolic activity could impair the structural and functional integrity of TLSs by modifying cell composition and disrupting intercellular signaling dynamics.

### 3.5 Tryptophan Metabolism Correlates with TLS Maturation via Immune-Related Transcriptional Programs

**Figure 5:**
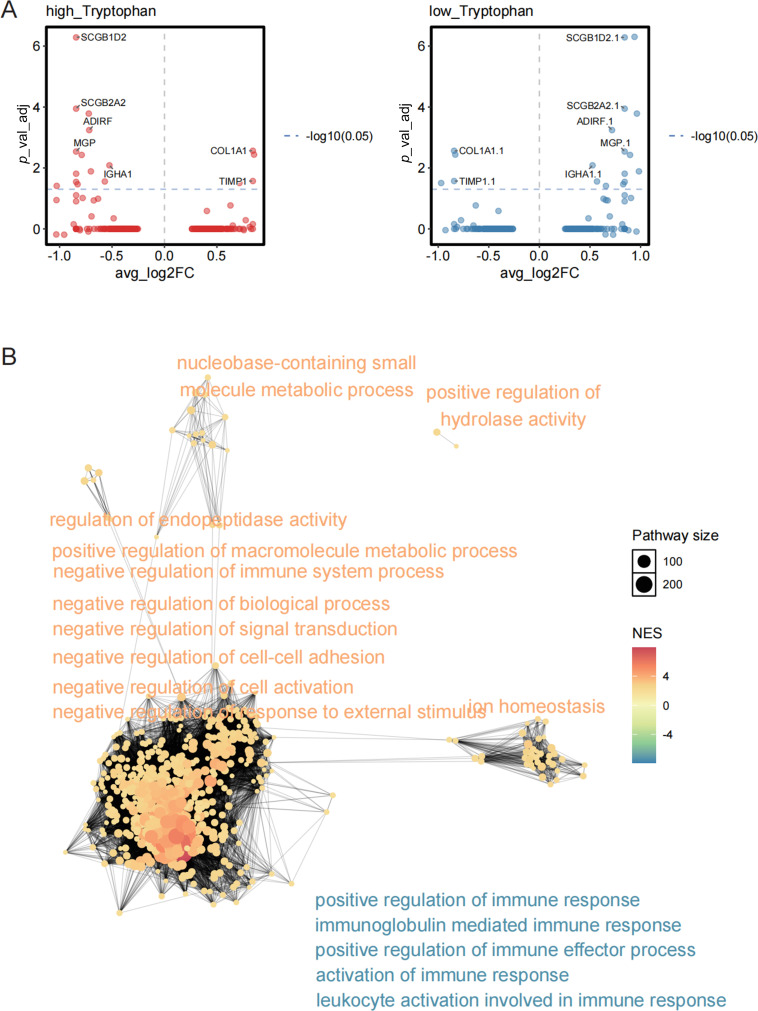
Differentially expressed genes and functional pathway enrichment analysis related to the maturity status of TLS. (**A**) The distribution of differentially expressed genes (DEGs) in the TLS high expression group (TLS-high, left) and the TLS low expression group (TLS-low, right), with the *x*-axis representing log2 Fold Change and the *y*-axis showing the adjusted *p* value (–log10 transformation). (**B**) Enrichment analysis of DEGs in the TLS high and low groups was conducted using the GO biological process gene set, and a functional network diagram was constructed. The size of the nodes represents the number of pathway-enriched genes, while the color indicates the enriched normalized enrichment score (NES)

To explore the molecular basis underlying TLS maturation status in the context of tryptophan metabolism, we stratified TLS regions into TLS-high and TLS-low groups and conducted differential gene expression analysis. The volcano plots revealed distinct transcriptomic signatures between the two groups, with multiple upregulated and downregulated genes exhibiting statistical significance (Fig. 5A). Notably, genes enriched in TLS-high regions were functionally annotated to immunologically relevant biological processes, such as B cell activation, antigen processing and presentation, and lymphocyte chemotaxis, whereas TLS-low regions displayed enrichment in processes related to cell stress response and metabolic regulation (Fig. 5B). Functional network analysis further highlighted a coordinated transcriptional program in TLS-high regions, with central nodes involving adaptive immune response, leukocyte-mediated immunity, and lymphoid organogenesis. These findings indicate that TLS maturity is associated with enhanced expression of immune-regulatory genes and suggest that tryptophan metabolism may exert its inhibitory effect by disrupting these functional pathways.

### 3.6 Spatial Coupling of Tryptophan Metabolism with TLS Signaling and High Endothelial Venule Networks

**Figure 6:**
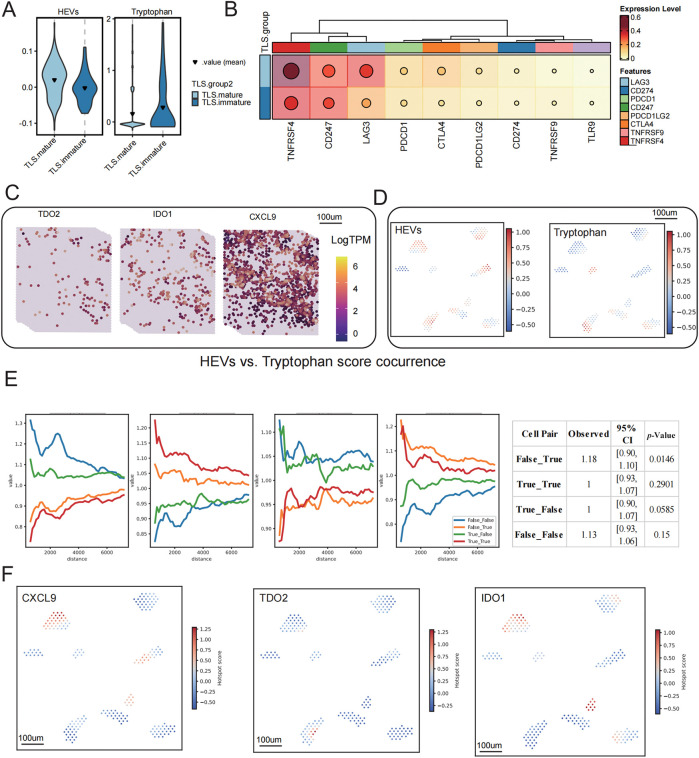

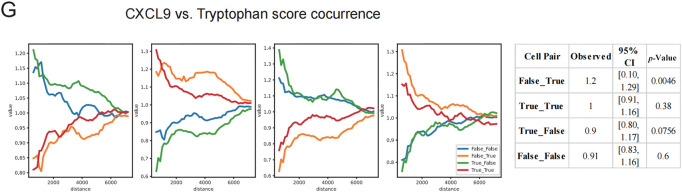
Spatial correlation analysis of TLS status with tryptophan metabolism, HEVs, and key molecules. (**A**) Differences in tryptophan metabolism scores and high endothelial venule (HEVs) scores across different TLS expression groups, displayed using a violin plot. (**B**) The expression of immune checkpoint-related genes among different TLS groups, with dot sizes representing average expression levels and color intensity indicating expression strength. (**C**) Spatial expression distribution maps of TDO2, IDO1, and CXCL9, visualized using LogTPM values. (**D**) Hotspot areas of HEVs and tryptophan metabolism scores, with high expression regions marked in red. (**E**) Spatial co-occurrence analysis of HEVs and tryptophan metabolism, constructing a distance model based on conditional probability to evaluate changes in co-occurrence probability at different distances. (**F**) Spatial hotspot maps of CXCL9, TDO2, and IDO1, revealing their local enrichment patterns within the tissue. (**G**) Analysis of the spatial co-occurrence relationship between CXCL9 and tryptophan metabolism scores, showing trend curves of distance changes under different co-occurrence states

To delineate the spatial associations between tryptophan metabolism, TLS development, and key immunological structures, we performed a comprehensive spatial correlation analysis. TLS-high regions exhibited significantly lower tryptophan metabolism and HEV scores compared to TLS-low areas (Fig. 6A), implicating a negative correlation between tryptophan catabolism and the presence of high endothelial venules (HEVs), an essential component of TLS maturation. Furthermore, a heatmap analysis revealed an overall downregulation of immune checkpoint genes such as PDCD1, CTLA4, and LAG3 in TLS-high regions, suggesting a less exhausted immune microenvironment (Fig. 6B).

Spatial transcriptomic visualization demonstrated that expression of TDO2 and IDO1, the key enzymes of tryptophan metabolism, was spatially segregated from CXCL9, a TLS-enriched chemokine (Fig. 6C). The spatial decoupling was further validated by hotspot mapping, which showed non-overlapping high-expression regions of HEV and tryptophan metabolism scores (Fig. 6D). A conditional co-occurrence distance model quantified this phenomenon, revealing that the spatial probability of co-localization between tryptophan metabolism and HEVs decreased as the distance between them decreased (Fig. 6E). Permutation-based significance testing (*n* = 1000) revealed that co-occurrence between *CXCL9*^−^*/TDO2*^+^ was significantly elevated beyond the null distribution (*p* = 0.0146), while *CXCL9*^+^*/TDO2*^+^ pairs did not differ significantly from random expectation (*p* = 0.29). This indicates a preferential enrichment of tryptophan metabolism signals away from TLS-high zones. Similar inverse spatial enrichment patterns were observed for CXCL9, TDO2, and IDO1 (Fig. 6F), and co-occurrence analysis confirmed a significant anti-correlation between CXCL9 and tryptophan metabolism in spatial proximity (Fig. 6G). Notably, CXCL9^−^/Tryptophan^+^ regions showed significantly stronger co-localization than expected by chance (*p* = 0.0046), while CXCL9^+^/Tryptophan^+^ proximity remained non-significant (*p* = 0.38). Collectively, these results indicate that heightened tryptophan metabolism spatially excludes key TLS signaling hubs and structural elements such as HEVs, providing a mechanistic explanation for the metabolic suppression of TLS maturation within the TIME.

### 3.7 Tryptophan Metabolism Negatively Regulates CXCL9 Expression and TLS Maturation Signals in Breast Cancer

**Figure 7:**
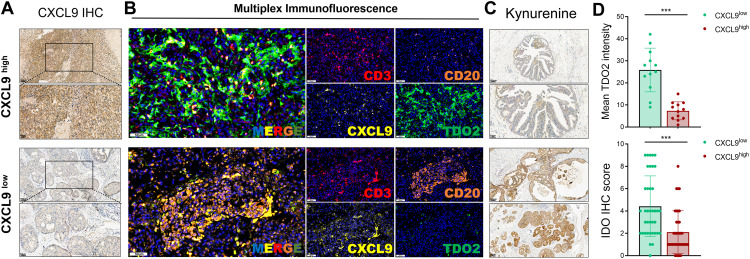
Validation of the inverse association between tryptophan metabolism and CXCL9 expression in human breast cancer tissues using IHC and multiplex immunofluorescence (mIF). (**A**) Representative IHC staining images and scoring of CXCL9 expression in 38 breast cancer specimens, which were subsequently stratified into CXCL9-high and CXCL9-low groups. Scale bars: 100 μm (high magnification) and 50 μm (low magnification) are shown in the lower left corner of each image. (**B**) Multiplex immunofluorescence staining of 12 breast cancer samples visualizing spatial co-localization and expression patterns of DAPI (nucleus), CD3 (T cells), CD20 (B cells), CXCL9, and TDO2 within the tumor microenvironment. Scale bars: 50 μm (high magnification) and 20 μm (low magnification) are shown in the lower left corner of each image. (**C**) Quantification of kynurenine pathway activity using IDO1 and INDO IHC scores across all 38 samples revealed an inverse correlation with CXCL9 expression. Scale bars: 100 μm (high magnification) and 50 μm (low magnification) are shown in the lower left corner of each image. (**D**) Samples in the CXCL9-low group exhibited significantly higher TDO2 fluorescence intensity and elevated tryptophan metabolism activity compared to the CXCL9-high group, validating a negative metabolic-immunological association *in situ* (^***^p < 0.001)

To validate the suppressive role of tryptophan metabolism on TLS maturation observed in transcriptomic analyses, we conducted IHC and mIF staining on breast cancer specimens with spatial TLS features. First, IHC was performed on 38 breast cancer tissue sections to assess CXCL9 expression, a key chemokine involved in TLS initiation and B cell recruitment. Based on IHC scores, samples were stratified into CXCL9-high and CXCL9-low groups (Fig. 7A). We then applied mIF staining in 12 representative samples to characterize the spatial distribution and co-expression of DAPI, CD3 (T cells), CD20 (B cells), CXCL9, and TDO2. In TLS-rich regions, robust CXCL9 expression colocalized with organized CD3^+^ and CD20^+^ cell clusters, indicating active TLS formation. Conversely, TLS-like structures in CXCL9-low tumors displayed disrupted spatial organization and increased TDO2 expression (Fig. 7B), suggesting an inhibitory effect of tryptophan metabolism on TLS integrity.

Furthermore, quantitative assessment of kynurenine pathway activity using IDO1 and INDO expression revealed a significant inverse correlation with CXCL9 levels across the 38-sample cohort (Fig. 7C). Notably, tumors in the CXCL9-low group exhibited significantly higher TDO2 fluorescence intensity and tryptophan metabolic activity (Fig. 7D), reinforcing the notion that elevated tryptophan catabolism may hinder TLS maturation by dampening CXCL9-enriched immune recruitment signals. These spatial and molecular findings confirm that high tryptophan metabolism within the tumor microenvironment correlates with a repressed TLS signature, highlighting a potential immunometabolic mechanism by which breast tumors evade effective local immune priming.

## 4 Discussion

In present study, we identified an association between elevated tryptophan metabolism and altered spatial immunoarchitecture in breast cancer, particularly in relation to impaired TLS maturation. By integrating bulk RNA-sequencing datasets, metabolic pathway activity scoring (e.g., GSVA), immune infiltration algorithms, single-cell RNA-seq (scRNA-seq), spatial transcriptomics, and multiplex immunofluorescence (mIF) validation, we found that tumors with high TDO2 expression were spatially depleted of TLS-rich regions and GC-like B cell populations, suggesting that TDO2-associated metabolic activity may influence the development of immune-supportive niches within the tumor microenvironment [36–38].

Tryptophan catabolism via the kynurenine pathway, predominantly catalyzed by indoleamine 2,3-dioxygenase 1 (IDO1) and tryptophan 2,3-dioxygenase (TDO2), has long been recognized as an important immunosuppressive mechanism in cancer [17,24,39,40]. Kynurenine accumulation contributes to immune tolerance by inducing regulatory T cells, which might inhibit cytotoxic T cell function, and modulating dendritic cell maturation. Rather than reiterating these established functions, our study provides additional insight by identifying an association between elevated TDO2 expression and suppressed TLS maturation. Specifically, tumors with high TDO2 levels exhibit lower CXCL9 expression, spatial exclusion of TLS-associated niches, and decreased signatures of B cell class switching, features that are consistent with less mature germinal center–like TLSs. These findings suggest a potential spatially constrained immunometabolic mechanism whereby TDO2 activity may be linked to impaired development of immune-supportive niches in breast cancer. While our observations point toward a functional relationship, they remain correlative, and future perturbation experiments will be required to establish direct mechanistic causality [40–42].

The presence and maturation status of TLS are increasingly recognized as key determinants of anti-tumor immunity, response to immune checkpoints blockade (ICB), and favorable prognosis in several malignancies, including breast cancer [43–45]. TLS are organized lymphoid aggregates that recapitulate the structure and function of secondary lymphoid organs, with mature TLS characterized by GC formation, follicular dendritic cells, and isotype-switched plasma cells [46]. Our spatial transcriptomic and colocalization analyses revealed that TDO2-expressing tumor regions displayed significantly reduced abundance of GC-like TLS and lower expression of markers associated with B cell maturation (e.g., AICDA, CD38, and IgG). Notably, TLS abundance and maturation were enriched in tumor areas with low TDO2 activity and high CXCL9 expression, highlighting the spatial opposition between immunosuppressive metabolism and immune-supportive niches [47,48]. This spatial antagonism underscores the potential of metabolic pathways to shape not only the immune cell composition but also the structural organization of immune infiltrates within tumors [49].

Our findings suggest that TDO2 may contribute to metabolic reprogramming potentially representing a key barrier to effective anti-tumor immunity by disrupting TLS maturation. These observations raise the possibility of metabolic inhibitors targeting TDO2 or downstream kynurenine signaling with immunotherapies aiming to augment TLS formation or function. While IDO1 inhibitors have previously failed in late-phase clinical trials (the ECHO-301/KEYNOTE-252 study) [50], TDO2 remains an underexplored target, and our data support its potential therapeutic value, particularly in TLS-deficient tumors [51]. Furthermore, TLS maturation status, assessed via spatial markers or B-cell isotype switching, may serve as a predictive biomarker for immunotherapy responsiveness [34,52,53].

In addition to the conceptual framework we propose, it is important to consider the translational feasibility of targeting TDO2. Previous attempts to inhibit the kynurenine pathway through IDO1 inhibitors, such as epacadostat, have not succeeded in late-stage clinical trials, underscoring the need to account for redundancy and compensation within this metabolic axis. Unlike IDO1, which is predominantly induced in stromal and immune cells, TDO2 is constitutively expressed in tumor cells and enriched in specific malignancies, including breast cancer. This distinction highlights TDO2 as a potentially more direct tumor-intrinsic target for reprogramming the immune landscape. Indeed, several small-molecule TDO2 inhibitors, such as 680C91 and LM10, have demonstrated preclinical efficacy in glioma and hepatocellular carcinoma models by reducing kynurenine accumulation and enhancing antitumor immunity. Furthermore, dual IDO1/TDO2 inhibitors are under active investigation as a strategy to overcome metabolic compensation within the kynurenine pathway. Although no TDO2-specific inhibitors have yet entered advanced clinical trials, our data provide a rationale for future studies to test whether inhibiting the TDO2–kynurenine–AhR axis can relieve local suppression of TLS maturation, restore B cell class switching, and improve antitumor immune responses [54,55].

This study has several limitations. A limitation of our study is that the causal relationship between TDO2 activity and TLS immaturity remains primarily inferential. Our conclusions are based on converging evidence from bulk and single-cell transcriptomics, spatial analyses, and multiplex immunofluorescence, all of which consistently indicate an inverse association between TDO2-driven tryptophan metabolism and TLS maturation. However, *in vivo* functional experiments, such as TDO2 overexpression or knockout in murine breast cancer models, were not performed. Given that TLS maturation critically depends on CXCL9/CXCL13 signaling and germinal center B cell differentiation, our findings extend these prior observations by suggesting that TDO2 may disrupt TLS integrity through suppression of B cell class switching and CXCL9 expression. Future work will involve the use of TDO2-knockout models or pharmacological inhibition studies to directly test this mechanism and validate its functional impact on TLS maturation *in vivo*. Second, our spatial analyses were constrained by the resolution of current spatial transcriptomic technologies, and the quantification of actual kynurenine concentrations was not directly assessed. Future studies should incorporate spatial metabolomics and *in situ* hybridization to further elucidate the biochemical and cellular interactions within TLS-associated regions. Finally, although this study observed a significant association between TDO2 activity and TLS formation, this relationship needs to be interpreted with caution. Both TDO2 expression levels and TLS density may be influenced by other tumor characteristics or microenvironmental features. For example, tumor subtype, inflammatory status, treatment exposure history, and stromal composition may all serve as confounding factors, influencing the association. Because this study data was derived from a retrospective analysis and relevant clinical variables were not fully controlled, these results should be considered hypothesis-generating rather than causal inference. Further prospective studies or mechanistic experiments are needed to further validate these observations.

## 5 Conclusion

In conclusion, our study delineates a novel immunometabolic mechanism through which TDO2-mediated tryptophan metabolism correlates with lower the formation and maturation of TLS in breast cancer. By inhibiting CXCL9 expression and B cell class switching, TDO2 activity hinders the establishment of immune-supportive niches, contributing to an immune-excluded tumor phenotype. These findings highlight TDO2 as a promising target for spatial immunomodulation and provide a foundation for developing TLS-based biomarkers and therapeutic strategies in breast cancer.

## Supplementary Materials

















## Data Availability

The raw data for this study were generated at the corresponding archives, further inquiries can be directed to the corresponding author.

## Supplementary Materials

The supplementary material is available online at https://www.techscience.com/doi/10.32604/or.2026.071122/s1.
