# Supplementary figures and images for: TDO2-Associated Tryptophan Metabolism Correlates with Impaired Tertiary Lymphoid Structure Maturation and Reduced B Cell Class Switching in Breast Cancer
Source: Oncol Res. 2026 Feb 24;34(3):26. doi: 10.32604/or.2026.071122 (PMC12963687; doi:10.32604/or.2026.071122)

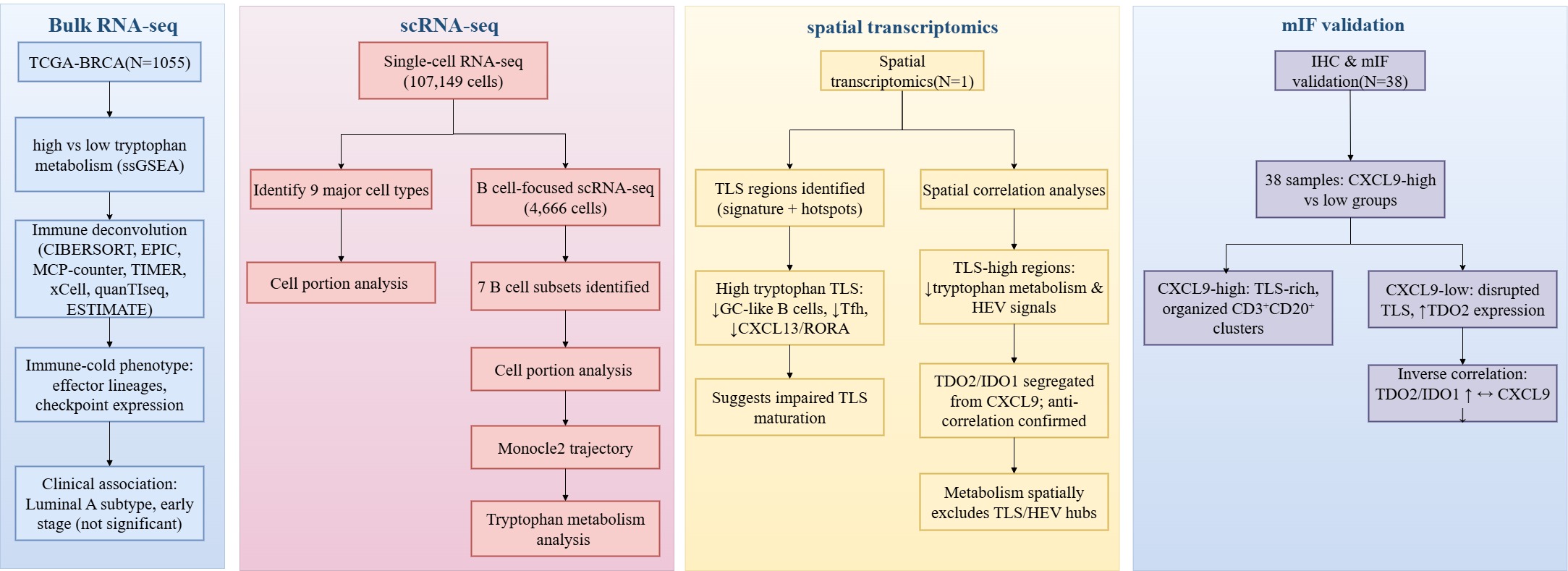

Supplement: Supplementary file 1 [file OncolRes-34-71122-s001.jpg]

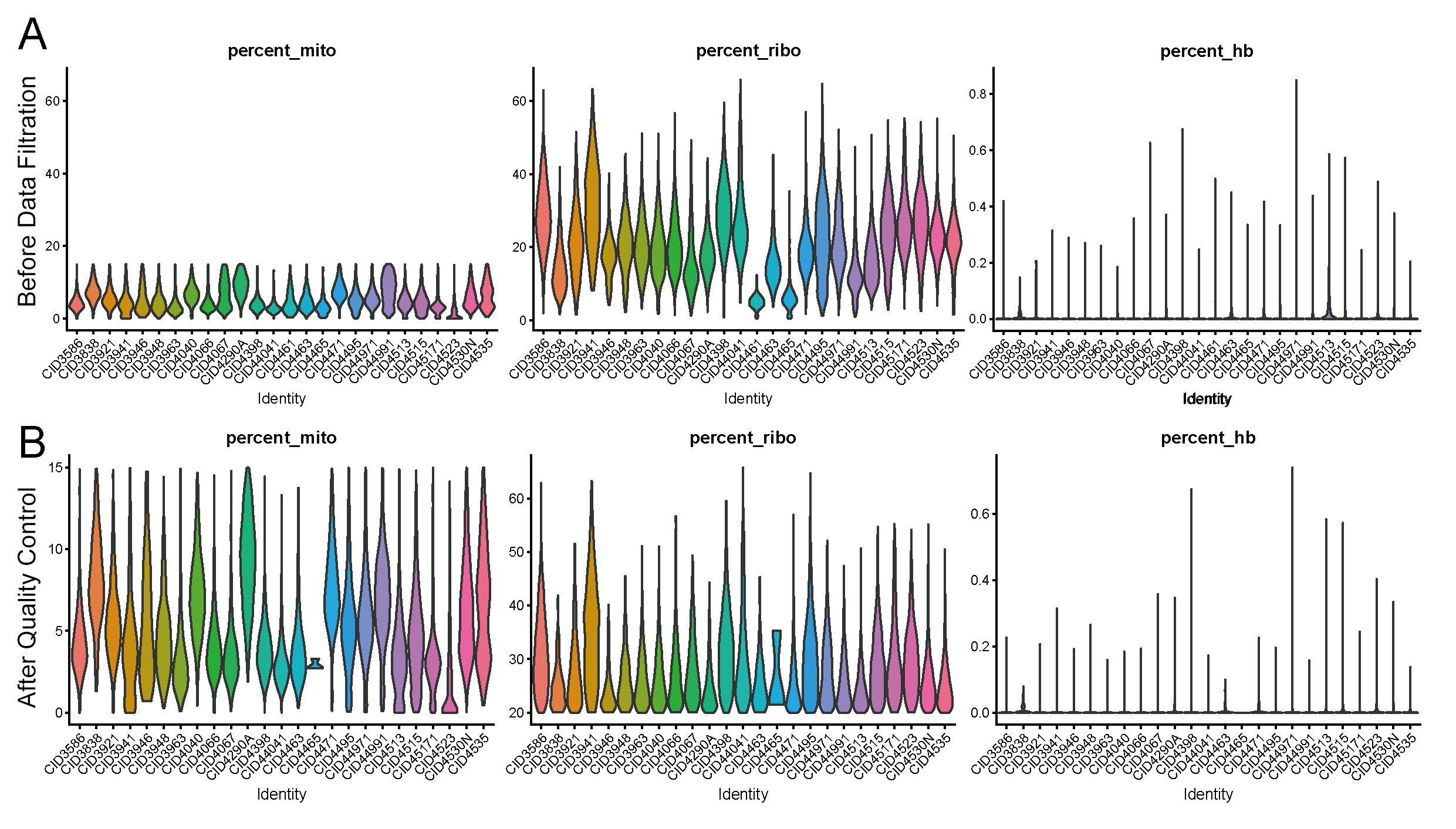

Supplement: Supplementary file 2 [file OncolRes-34-71122-s002.tif]

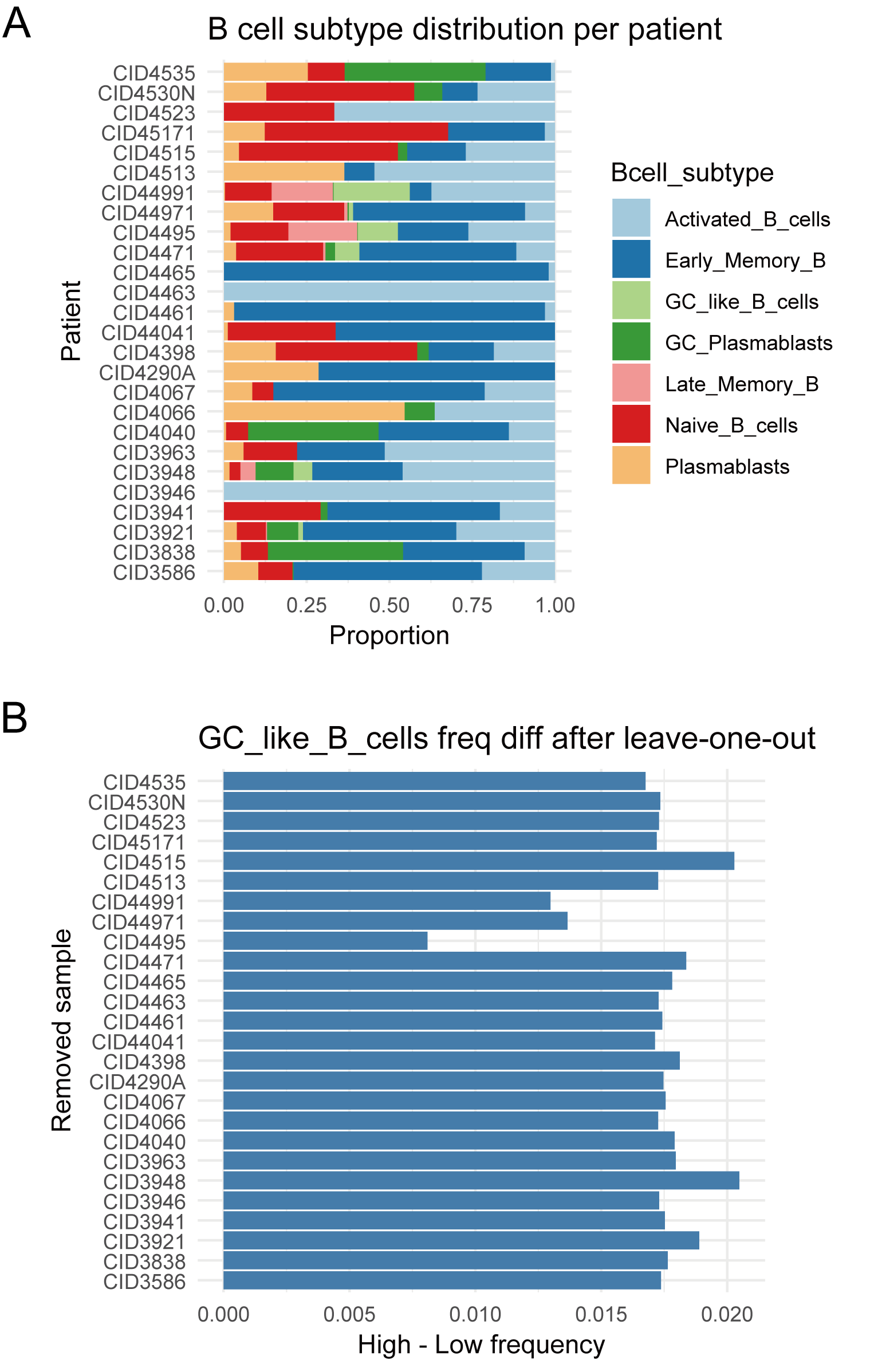

Supplement: Supplementary file 3 [file OncolRes-34-71122-s003.tif]
