# Supplementary material for: TDO2-Associated Tryptophan Metabolism Correlates with Impaired Tertiary Lymphoid Structure Maturation and Reduced B Cell Class Switching in Breast Cancer
Source: Oncol Res. 2026 Feb 24;34(3):26. doi: 10.32604/or.2026.071122 (PMC12963687; doi:10.32604/or.2026.071122)
Supplement: Supplementary file 4 [file OncolRes-34-71122-s004.docx]

**Supplementary table 1.** Cell counts across the 26 samples.

| **Sample ID** | **Cell count** |
| --- | --- |
| **CID3586** | 6113 |
| **CID3838** | 2271 |
| **CID3921** | 2870 |
| **CID3941** | 575 |
| **CID3946** | 568 |
| **CID3948** | 2236 |
| **CID3963** | 3439 |
| **CID4040** | 2470 |
| **CID4066** | 5255 |
| **CID4067** | 3356 |
| **CID4290A** | 4994 |
| **CID4398** | 4381 |
| **CID44041** | 2125 |
| **CID4461** | 477 |
| **CID4463** | 1094 |
| **CID4465** | 1157 |
| **CID4471** | 8199 |
| **CID4495** | 7729 |
| **CID44971** | 7731 |
| **CID44991** | 6078 |
| **CID4513** | 5579 |
| **CID4515** | 4086 |
| **CID45171** | 2328 |
| **CID4523** | 1660 |
| **CID4530N** | 4264 |
| **CID4535** | 3497 |
