# Supplementary material for: TDO2-Associated Tryptophan Metabolism Correlates with Impaired Tertiary Lymphoid Structure Maturation and Reduced B Cell Class Switching in Breast Cancer
Source: Oncol Res. 2026 Feb 24;34(3):26. doi: 10.32604/or.2026.071122 (PMC12963687; doi:10.32604/or.2026.071122)
Supplement: Supplementary file 5 [file OncolRes-34-71122-s005.docx]

| **Environment** | **Package** | **Version** |
| --- | --- | --- |
| **R 4.2.1 (Bioconductor 3.18)** | Seurat | 4.4.0 |
|  | SeuratObject | 4.1.4 |
|  | Matrix | 1.6.5 |
|  | ggplot2 | 3.5.1 |
|  | dplyr | 1.1.4 |
|  | data.table | 1.15.4 |
|  | patchwork | 1.2.0 |
|  | cowplot | 1.1.3 |
|  | clustree | 0.5.1 |
|  | future | 1.33.2 |
|  | Rcpp | 1.0.13 |
|  | harmony | 1.2.0 |
|  | DoubletFinder | v2.0.3 |
|  | GSVA | 1.50.0 |
|  | fgsea | 1.28.0 |
|  | clusterProfiler | 4.10.0 |
|  | org.Hs.eg.db | 3.18.0 |
|  | SingleR | 2.8.0 |
|  | celldex | 1.14.0 |
|  | spacexr (RCTD) | 1.3.0 |
|  | monocle | 2.24.1 |
|  | IOBR | 0.99.9 |
|  | ggsci | 3.0.0 |
|  | ggpubr | 0.6.0 |
|  | ComplexHeatmap | 2.16.0 |
| **Python 3.7.0** | scanpy | 1.9.8 |
|  | squidpy | 1.2.2 |
|  | hotspot | 1.0.0 |
|  | numpy | 1.26.4 |
|  | pandas | 2.2.2 |
|  | scipy | 1.14.1 |
|  | matplotlib | 3.9.2 |
|  | pynndescent | 0.5.13 |
|  | Pillow | 10.4.0 |

**Supplementary table 2.** Software and version used in this study.
